# TLR7 Stimulation With Imiquimod Induces Selective Autophagy and Controls *Mycobacterium tuberculosis* Growth in Mouse Macrophages

**DOI:** 10.3389/fmicb.2020.01684

**Published:** 2020-07-17

**Authors:** Hyo-Ji Lee, Su-Jin Kang, Yunseo Woo, Tae-Wook Hahn, Hyun-Jeong Ko, Yu-Jin Jung

**Affiliations:** ^1^Department of Biological Sciences, Kangwon National University, Chuncheon, South Korea; ^2^Institute of Life Sciences, Kangwon National University, Chuncheon, South Korea; ^3^College of Veterinary Medicine, Kangwon National University, Chuncheon, South Korea; ^4^College of Pharmacy, Kangwon National University, Chuncheon, South Korea

**Keywords:** autophagosome, mycobactericidal activity, imiquimod (IMQ), mitophagy, *Mycobacterium tuberculosis*, toll-like receptor 7 (TLR7)

## Abstract

Autophagy is a lysosomal self-digestion pathway that maintains internal homeostasis inside cells and critical process by which the innate immune system eliminates intracellular bacteria. In this study, we showed that stimulation of toll-like receptor 7 (TLR7) with imiquimod (IMQ) triggered autophagic cell death in macrophages by enhancing the generation of reactive oxygen species (ROS) via the p38- or MEK/ERK1/2-mediated signaling pathway in the early phase. IMQ significantly increased mitochondrial ROS and targeted autophagosomes to the mitochondria. Stimulation of TLR7 with IMQ enhanced the expression of BNIP3, which was localized to mitochondria and interacted with beclin-1, leading to mitophagy. In addition, IMQ substantially induced NO production through the GSK-3β-mediated signaling pathway, which led to autophagy in the late stage. We further examined whether the induction of autophagy by IMQ effectively eliminated intracellular microbes. Macrophages were infected with a virulent *Mycobacterium tuberculosis* (Mtb) strain, H37Rv, and then treated with IMQ. IMQ suppressed intracellular Mtb growth by inducing autophagy in a dose-dependent manner and increased NO production. Inhibition of autophagy using 3-methyladenine (3-MA) prevented autophagosome formation and control of intracellular Mtb growth in macrophages. These findings revealed a novel mechanism by which IMQ induces selective autophagy to promote intracellular killing machinery against Mtb infection in macrophages.

## Introduction

Autophagy is conserved in virtually all eukaryotic cells and is a fundamental cellular homeostatic process that mediates the degradation of long-lived proteins, cellular organelles, and invading pathogens, including *Mycobacterium tuberculosis* (Mtb), *Shigella flexneri*, and *Salmonella typhimurium* (King, 2012; Kimmey and Stallings, 2016; Yin et al., 2016). During autophagy, cytoplasmic organelles are surrounded by a phagophore or isolation membrane to form an autophagosome, a double-membraned vesicle that sequesters cytoplasmic material (Liu et al., 2013; Ryter et al., 2013). The autophagosome then fuses with a lysosome to degrade its contents. The autophagic process is negatively regulated by mammalian target of rapamycin (mTOR), and inhibition of mTOR initiates the recruitment of beclin-1 and other “autophagy-related (ATG) proteins,” such as the ATG5-ATG12 complex, ATG16 and LC3 (ATG8) (Deretic, 2012; Shibutani et al., 2015). Formation of the autophagosome is dependent on the conversion of microtubule-associated protein light chain 3 (LC3-I) to the lipidated form, LC3-II, which is a common indicator of autophagy (Kliosnky, 2016).

Autophagy is universally considered to be a nonselective degradation system. However, current evidence strongly suggests that autophagy of mitochondria, endoplasmic reticulum (ER), ribosomes, peroxisomes, and other organelles is selective (Gatica et al., 2018). The selective autophagy of mitochondria, termed mitophagy, is a quality control mechanism in which damaged or unwanted mitochondria are eliminated by autophagosomes (Kamber et al., 2015; Zaffagnini and Martens, 2016). In addition to its role in the control of damaged mitochondria, mitophagy has been associated with diverse human diseases, such as Parkinson’s disease, cancer and viral infection, in several studies (Khan et al., 2015; Gao et al., 2017; Kulikov et al., 2017). Recently, receptor interacting protein kinase 2 (RIPK2) was shown to specifically regulate mitophagy and the accumulation of damaged mitochondria due to downregulation of phosphorylated ULK1, which is a critical autophagy protein, during influenza A virus (IAV) infection (Lupfer et al., 2013). Moreover, mitophagy was reported to be suppressed by the hepatitis C virus (HCV) core protein through interaction with the mitophagy-related protein parkin (Hara et al., 2014). Although mitophagy is associated with resistance to viral infection, the putative roles of mitophagy during bacterial infection are still unclear.

Toll-like receptors (TLRs) are one of the best characterized pattern recognition receptors (PRRs) for the detection of various pathogen-associated molecular patterns (PAMPs), including lipids, lipoproteins, proteins, and nucleic acids, which are broadly expressed by different groups of microorganisms and induce innate immune responses (Kawasaki and Kawai, 2014; De Nardo, 2015). Our previous study demonstrated that stimulation with a TLR7 agonist enhances anticancer effects via autophagic cell death during radiotherapy for melanoma, suggesting that TLR7 agonists can induce autophagic cell death (Cho et al., 2017). The induction of autophagy via TLR7 was shown to depend on MyD88 expression and eliminate intracellular microbes, such as intracellular Bacillus Calmette-Guérin (BCG), in mouse macrophages (Delgado et al., 2008). A recent study also showed that the expression of TLR7 was upregulated and that stimulation of TLR7 with ssRNA inhibited intracellular Mtb survival through autophagy in Mtb-infected macrophages (Bao et al., 2017). However, little is known about the intracellular mechanisms by which stimulation of TLR7 modulates autophagy in macrophages. In this study, we found that stimulation of TLR7 with imiquimod (IMQ) led to autophagic cell death by increasing nitric oxide (NO) production via early MEK/ERK- and late GSK-3β-mediated signaling pathways in macrophages. In addition, IMQ significantly increased mitochondrial reactive oxygen species (ROS) and targeted autophagosomes to mitochondria, leading to mitophagy by enhancing the interaction between BNIP3 and beclin-1. Stimulation of TLR7 with IMQ strongly suppressed intracellular Mtb survival by inducing autophagy in a dose-dependent manner and increased NO production. Thus, our study enhances the understanding of the intracellular mechanism of TLR7-induced autophagy in Mtb infection. These results also indicated that IMQ could potentially be incorporated into effective treatment strategies to enhance a selective autophagy during Mtb infection.

## Materials and Methods

### Cell Culture

A mouse macrophage cell line, Raw264.7, and human monocytic leukemia cell line, THP-1, were purchased from the American Type Culture Collection (ATCC) and maintained in RPMI-1640 (Lonza, Walkersville, United States) culture medium containing 10% heat-inactivated fetal bovine serum (FBS; Lonza, Walkersville, United States), penicillin (10,000 U/L), and 100 μg/L streptomycin (Gibco-BRL, Gaithersburg, United States) at 37°C and 5% CO_2_. THP-1 cells were differentiated into macrophages by treatment with PMA (20 ng/mL) for 2 days.

### Reagents

All TLR7 agonists were purchased from InvivoGen (San Diego, United States), including gardiquimod (GDQ; 2.4μg/mL) and IMQ (2 μg/mL). 3-MA (10 mM), necrostatin-1 (Nec-1; 50 μM), and the MEK1 inhibitor PD98059 (20 μM) were purchased from Sigma-Aldrich (St. Louis, United States). A pancaspase inhibitor, Z-VAD-FMK (10 μM), was purchased from Promega (Madison, United States). A PI3K inhibitor, wortmannin (20 μM), and a MEK1/2 inhibitor, U0126 (10 μM), were purchased from Cell Signaling Technologies (Danvers, United States). A GSK-3 inhibitor, SB216763 (10 μM), was purchased from TOCRIS (Ellisville, Missouri, United States).

### Mtb Infection

The Mtb strain H37Rv, a laboratory-adapted virulent strain, was used for all infections. Mouse macrophages (4 × 10^4^ per well) were seeded in six-well cell culture plates (SPL Lifesciences, Pocheon, South Korea) for 4 days at 37°C with 5% CO_2_. Nonadherent cells were washed away using PBS, and then, the cells were infected with Mtb at a multiplicity of infection (MOI) of 0.1, 1, or 10. After 4 h, extracellular bacteria were removed with PBS. Infected macrophages were lysed with 0.1% saponin for 10 min and inoculated in Middlebrook 7H10 agar containing 10% OADC to identify the growth of intracellular bacteria. Colony-forming units (CFUs) were determined at 21 days after inoculation.

### siRNA Transfection

The siRNA target sequence for mouse GSK-3β, Atg7 or BNIP3 was purchased from Santa Cruz Biotechnology. siRNA transfection was performed as previously described (Lee et al., 2015). Raw264.7 cells were transfected with siRNA using Lipofectamine 2000 (Invitrogen, United States) according to the manufacturer’s instructions. siRNA was used at a final concentration of 60 pM.

### WST-1 Proliferation Assay

Cell proliferation assays were performed using WST-1 reagent (TaKaRa, Otsu, Japan). Raw264.7 cells (5 × 10^4^ per well) were plated in 96-well plates and incubated for 2 days. After 2 days, the cells were treated with GDQ or IMQ at different concentrations. In addition, the cells were pretreated with 3-MA (10 mM) and then treated with IMQ. At each time point, WST-1 reagent was added to each well, followed by incubation for 4 h. The absorbance at 450 nm was measured using an enzyme-linked immunosorbent assay (ELISA) reader (Biotek Instruments Inc., Winooski, United States).

### Lactate Dehydrogenase (LDH) Release Assay

Lactate dehydrogenase release in supernatants was determined to evaluate cell viability. LDH release in culture supernatants was measured using a nonradioactive LDH assay kit (Promega, Madison, WI, United States) according to the manufacturer’s instructions. The released formazan was quantified by measuring the absorbance at 490 nm using an ELISA reader (Biotek Instruments Inc., Winooski, United States).

### Western Blot Assay

Whole-cell extracts were isolated using RIPA buffer [10 mM Tris-HCl (pH 8.0), 1 mM EDTA, 140 mM NaCl, 0.1% deoxycholate, 0.1% SDS, 0.1% Triton X-100] supplemented with protease inhibitors (Calbiochem, Darmstadt, Germany). The concentration of the extracted proteins was determined using a Bradford Assay Kit (Bio-Rad, Hercules, United States). The extracted protein was loaded onto a 10 or 15% SDS-PAGE gel, followed by transfer to a PVDF transfer membrane (Millipore, Bedford, United States). Western blot assays were performed as previously described (Lee et al., 2015). Anti-α-tubulin, anti-actin, anti-phospho-GSK-3β, and siGSK-3β were purchased from Santa Cruz (Delaware Avenue, United States). Anti-iNOS, anti-LC3B, anti-phospho-MEK1/2, anti-MEK1/2, anti-phospho-ERK, anti-ERK, anti-PI3K, anti-GSK-3β, anti-phospho-p70S6K, and anti-p70S6K were purchased from Cell Signaling Technologies (Danvers, United States). Anti-BNIP3 was purchased from Abcam (Cambridge, MA, United States). All blots were detected in cropped membranes according to protein size. Blots were treated with ECL (Animal Genetics Inc., Suwon, South Korea) and exposed to Kodak XAR film in the darkroom.

### Immunoprecipitation Analysis

Raw264.7 cells were treated with IMQ for the indicated times and washed with PBS. Then, cells were lysed with IP lysis buffer (50 mM Tris-HCl, pH 7.5, 250 mM NaCl, and 0.5% NP-40) containing protease inhibitor and centrifuged at 13,200 RPM for 10 min. Supernatants were incubated with anti-beclin-1 at 4°C for 2 h on a rotator. The antibody complexes were isolated with protein A/G agarose beads (Millipore, United States). After centrifugation, the mixture was washed using IP lysis buffer three times, mixed with SDS sample buffer and boiled to dissociate protein A/G agarose beads bound to immune complexes. Samples were analyzed by Western blot assays.

### RNA Isolation and RT-PCR

Total RNA was obtained using the RNeasy mini kit according to the manufacturer’s procedure (Qiagen, Valencia, CA, United States). Total RNA was converted to cDNA using M-MLV RT (Enzynomics, Daejeon, South Korea), RNase inhibitor and random hexamers (TaKaRa, Otsu, Japan). Negative control reactions were performed as described above, with omission of the enzyme or cDNA. Standard cycling was performed with 35 cycles of 95, 55, and 72°C steps of 30 s each. The PCR products were analyzed by electrophoresis on a 1.5% agarose gel in Tris acetate-EDTA buffer [TAE; Tris base, glacial acetic acid, 0.5 M EDTA (pH 8.0)]. Glyceraldehyde-3-phosphate dehydrogenase (GAPDH) and β-actin were used as internal controls. The primer sequences used for RT-PCR were as follows: mouse BNIP3: forward, 5′-AGAACCTGCAGGGCTCCTGG-3′ and reverse, 5′-GAAGTTGTCAGACGCCTTCC-3′; mouse GAPDH: forward, 5′-TGCTGATGTCGTGGAGTCT-3′ and reverse, 5′-AATGGGAGTTGCTGTTGAAGTC-3′.

### CFU Assays

Raw264.7 mouse macrophages were infected with H37Rv for 4 h and then washed with PBS twice to remove extracellular bacteria. After cell lysis with 0.1% saponin (Sigma-Aldrich, St. Louis, United States), samples were diluted 1/10, 1/100, 1/1,000, 1/10,000, and 1/100,000 and transplanted to Middlebrook 7H10 agar containing 10% OADC. After inoculation, colonies were counted at 21 days.

### Immunofluorescence Analysis

For immunostaining, mouse macrophages (5 × 10^4^ per well) were seeded on 18 mm diameter round glass coverslips in 12-well culture plates and incubated for 2 days. Immunofluorescence analysis was performed as previously described (Lee et al., 2016). Briefly, cells were treated with IMQ or infected with Mtb for the indicated time, and the cells were then fixed with 4% paraformaldehyde at room temperature for 15 min. After permeabilization with 0.2% Triton X-100, the samples were stained with primary antibody and FITC anti-rabbit secondary antibody (Jackson ImmunoResearch, West Grove, United States). Nuclei were counterstained with 4′-6-diamidino-2-phenylindole (DAPI, Sigma-Aldrich, St. Louis, United States). For analysis of the induction of mitophagy, cells were treated with IMQ, incubated with MitoTracker Red^TM^ (100 nM, Molecular Probes, United States) for 30 min and fixed with 4% paraformaldehyde at room temperature for 15 min. Then, cells were permeabilized with 0.2% Triton X-100 and stained with anti-LC3 (MBL, Nagoya, Japan), followed by a fluorophore-conjugated secondary antibody. To determine the localization of BNIP3, cells were treated with IMQ, fixed and permeabilized as described above. Then, the cells were stained with anti-BNIP3 followed by a fluorophore-conjugated secondary antibody (anti-mouse IgG Alexa 488). Cover slides were mounted with Fluoromount-G^TM^ and monitored by confocal microscopy (FV1000 SPD, Olympus, Tokyo, Japan). The percentage of autophagic flux was quantified by the number of LC3^+^ puncta-containing cells relative to the total number of cells in five randomly selected regions on each slide. The percentage of mitophagy was quantified by calculating the number of cells exhibiting the overlapping signal of LC3^+^ puncta with MitoTracker Red relative to the total number of cells on each slide. BNIP3 overlap with MitoTracker Red was measured as the percentage of cells expressing BNIP3 and MitoTracker Red relative to the number of total cells. For quantitative analysis, five regions on each slide were randomly selected. All quantitative data were calculated from 30 to 50 cells in the acquired images and reported as the mean ± SD. All experiments were performed in triplicate and repeated three times.

### Transmission Electron Microscopy (TEM)

For observation under a transmission electron microscope, cells were fixed in 3% formaldehyde and 2% glutaraldehyde for 1 h after stimulation with IMQ for 30 h. Each sample was dehydrated with increasing concentrations of ethanol, gradually adsorbed with Epon-Araldite resin and inserted in straight resin. The samples were heated at 80°C for 24 h to harden. After ultrathin sections (70–80 nm) were made using an ultramicrotome, the sections were stained with uranyl acetate and lead citrate and observed by TEM. This observation was performed at the Korea Basic Science Institute (KBSI, Chuncheon, South Korea).

### Detection of Nitric Oxide (NO) Production

NO production was detected as previously described (Lee et al., 2016). NO production was measured in culture supernatants using an NO detection kit (Intron Bio-technology Inc., Seoul, South Korea) according to the manufacturer’s procedure. Raw264.7 cells were treated with IMQ or infected with H37Rv, and then, the cell supernatant was collected at the indicated time points. The nitrite content in the culture supernatant was measured by absorbance at 540 nm using a microplate reader (Biotek Instruments Inc., Winooski, United States) and calculated from a standard curve of purified nitrite.

### Enzyme-Linked Immunosorbent Assay

Enzyme-linked immunosorbent assays were carried out with cell culture supernatant according to the manufacturer’s instructions. The absorbance of the plate was measured at 405 nm using an ELISA reader (Biotek Instruments Inc., Winooski, United States). Recombinant murine TNF-α and IL-10 were used as standards.

### Intracellular ROS Measurement

Intracellular ROS measurement was performed using 2′,7′-dichlorofluorescin diacetate (DCF-DA). In brief, cells were treated with IMQ or infected with H37Rv and loaded with 5 μM DCF-DA for 20 min at 37°C and 5% CO_2_. Cover slides were washed two times with PBS, mounted with Fluoromount-G^TM^ and monitored by confocal microscopy. For analysis of mitochondrial ROS production, cells were treated with IMQ in the presence or absence of apocynin. Cells were washed with PBS and incubated with MitoSOX Red (Molecular Probes, United States) for 30 min. For the quantification of mitochondrial ROS, the overlapping signal of MitoTracker Red^TM^ and DCF fluorescence was measured at 40× magnification. Merged images were generated and quantified using FV10-ASC software provided by Olympus. For flow cytometry, the cells were treated as described above and analyzed using a FACSCalibur flow cytometer (BD Bioscience). All FACS data were analyzed using CellQuest software.

### Statistical Analysis

For statistical analysis, data were obtained from three independent experiments. Statistical significance was determined by one-way ANOVA followed by Tukey’s *post hoc* test or two-way ANOVA followed by Bonferroni’s *post hoc* test using GraphPad Prism 5 (GraphPad software). Differences were considered significant at ^∗^*p* < 0.05; ^∗∗^*p* < 0.01, and ^∗∗∗^*p* < 0.001; ns was used to indicate that a difference was not significant (*p* > 0.05).

## Results

### IMQ Induces Autophagic Cell Death in Macrophages

**FIGURE 1:**
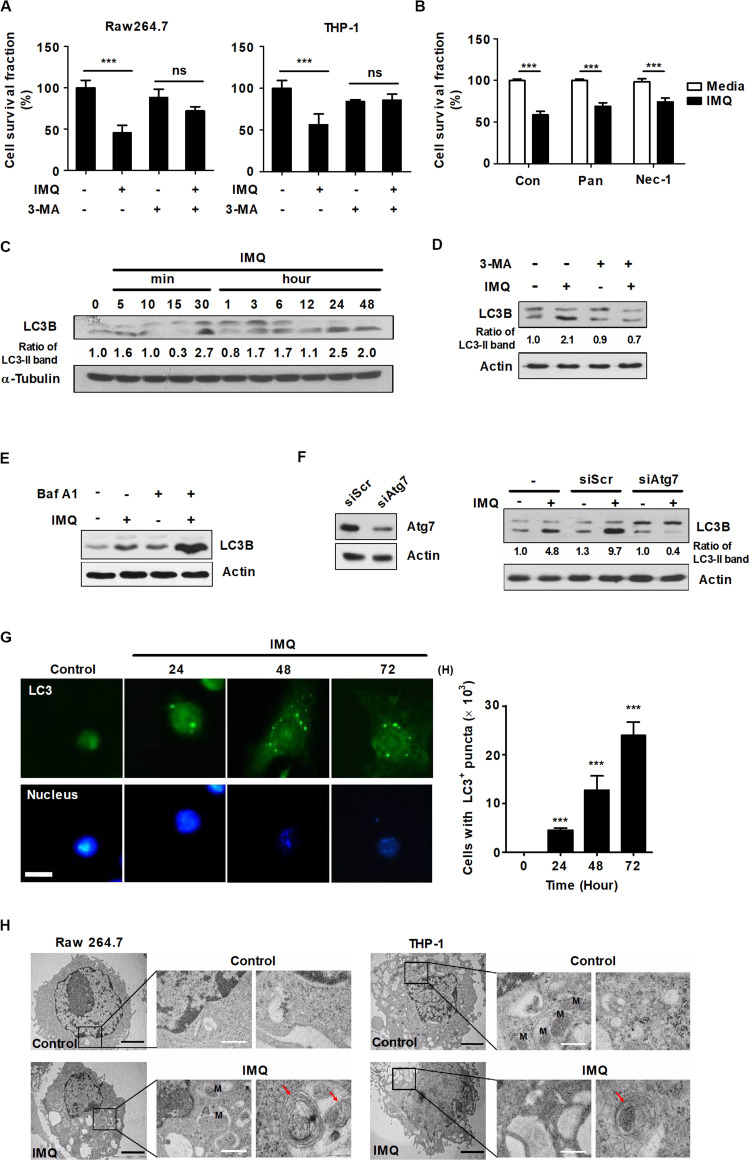
IMQ induces autophagy in macrophages. **(A)** Raw264.7 or THP-1 cells were treated with IMQ (2 μg/mL) for 48 h in the presence or absence of 3-MA (10 mM for 2 h). **(B)** Raw264.7 cells were pretreated with necrostatin-1 (Nec-1; 50 μM for 1 h) or z-VAD-FMK (Pan; 10 μM for 2 h) and treated with IMQ. **(A,B)** Cell survival was assessed by WST-1 assays. **(C,D)** Western blots were performed with antibodies against LC3, α-tubulin or actin in Raw264.7 cells. **(E)** Raw264.7 cells were pretreated with bafilomycin A1 and then treated with IMQ. Protein levels of LC3B and actin were detected by a Western blot assay. **(F)** Raw264.7 cells were transfected with scrambled siRNA (siScr) or siRNA targeting Atg7 and then treated with IMQ (2 μg/mL) for 48 h. Protein levels of LC3B, actin and Atg7 were detected by a Western blot assay. **(G)** Raw264.7 cells were incubated with IMQ (2 μg/mL) for the indicated times, and intracellular LC3 (green) was monitored. The nucleus was stained with DAPI (blue). Left panel: representative immunofluorescence images; scale bar 5 μm. Right panel: quantitative analysis of the percentages of cells with LC3^+^ puncta. **(H)** Multilayer membranes were observed by TEM. M, mitochondria, autophagic vacuoles; red arrows, black scale bar; 2 μm, white scale bar; 1 μm. **(A–G)** Data are the means ± s.d. of three technical replicates and are representative of at least three independent experiments. **(H)** Images are representative of at least three independent experiments. Statistical significance is indicated as ***p < 0.001 and ns, not significant (*p* > 0.05).

TLR7 is expressed in the endosome of macrophages and recognizes synthesized imidazoquinoline amine derivatives and guanosine analogs as well as viral ssRNA (Akinbobuyi et al., 2016). We verified the expression of TLR7 in mouse bone marrow-derived macrophages (BMDMs), splenocytes, Raw264.7 cells and THP-1 cells, a human monocytic cell line, by RT-PCR (Supplementary Figure S1A). We then assessed the effects of commercial agonists of TLR7 on cell growth by WST-1 assays. The viability of macrophages was decreased in a dose-dependent manner after treatment with IMQ but not GDQ, another agonist of TLR7 (Supplementary Figures S1B,C). The formation of an intracellular vesicular structure in the cytoplasm was detected in the IMQ-treated cells (Supplementary Figure S1D). To elucidate the mechanisms underlying IMQ-induced cell death, we pretreated cells with 3-MA as an autophagy inhibitor, Z-VAD-fmk (Pan) as a pancaspase inhibitor or Nec-1 as a necrosis inhibitor and then stimulated the cells with IMQ. Intriguingly, pretreatment with 3-MA provided protection from IMQ-induced cell death in both Raw264.7 mouse macrophages and THP-1 human macrophages (Figure 1A); however, Pan and Nec-1 did not affect the viability of Raw264.7 cells (Figure 1B). We then performed an additional cell death assay using an LDH assay kit to demonstrate that IMQ induces autophagic cell death in macrophages. Treatment with IMQ significantly enhanced LDH release; however, this change was reversed in the presence of bafilomycin A or in Atg7-deficient macrophages (Supplementary Figures S1E,F), suggesting that IMQ induces autophagic cell death. Since the extent of conversion of LC3-I to LC3-II is the hallmark of autophagy and correlated with the level of autophagy (Kliosnky, 2016; Zhang Z. et al., 2016), LC3-I-to-LC3-II conversion was detected by Western blot analysis. The conversion of LC3-I to LC3-II increased in IMQ-stimulated cells at late time points (12–48 h) as well as at an early time point (30 min) (Figure 1C), whereas it decreased after inhibition of autophagy using 3-MA (Figure 1D). In addition, pretreatment with the lysosome inhibitor bafilomycin A1 also induced the accumulation of a large amount of LC3-II in IMQ-treated cells (Figure 1E). We further investigated whether IMQ induced autophagic cell death in macrophages. Cells were transfected with either siAtg7 or scrambled RNA. IMQ led to a significant increase in the conversion of LC3-II, which was decreased in the absence of Atg7 (Figure 1F). We further performed LC3 immunofluorescence staining to monitor autophagosome accumulation. As shown in Figure 1G, compared to the untreated control, treatment with IMQ significantly enhanced the formation of endogenous LC3-positive puncta in a time-dependent manner (24–72 h). We obtained further evidence for IMQ-induced autophagy from TEM and observed multiple autophagosome-like vacuoles with double-membrane structures in IMQ-treated Raw264.7 and THP-1 cells (Figure 1H). Taken together, these data indicated that IMQ induced autophagy in macrophages.

### IMQ-Induced NO Generation Is Mediated by the MEK/ERK1/2 and GSK-3β Pathways

**FIGURE 2:**
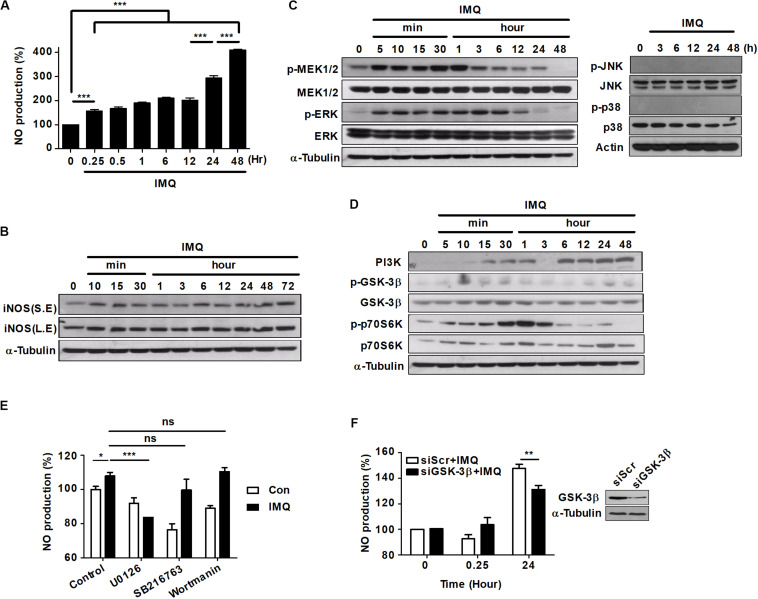
NO production is induced at a late time point after IMQ treatment in macrophages. **(A)** Raw264.7 cells were treated with IMQ (2 μg/mL) for the indicated times. NO production was measured in culture supernatants using an NO detection kit. **(B)** Western blot analysis using antibodies against iNOS and α-tubulin. S.E., short-exposure; L.E., long-exposure. **(C,D)** Total cell lysates were assessed by Western blot analysis for the detection of **(C)** phospho-MEK1/2, total MEK1/2, phospho-ERK, total ERK, **(D)** PI3K, phospho-GSK-3β (Ser9), total GSK-3β, phospho-p70S6K, and total p70S6K. α-Tubulin was utilized as the loading control. **(E)** Raw264.7 cells were pretreated with U0126, SB216763, or wortmannin for 1 h before treatment with IMQ. Culture supernatants were used for the detection of NO production. **(F)** NO production was detected in culture supernatants of Raw264.7 cells that were transfected with a specific siRNA for GSK-3β (siGSK-3β). Western blot assays were performed to assess transfection efficiency. All experiments were carried out in three independent experiments. Statistical significance is indicated as *p < 0.05, **p < 0.01, ***p < 0.001, and ns, not significant (*p* > 0.05).

Recently, several groups reported that excess ROS and NO function as signaling molecules in a variety of intracellular processes, and they might also contribute to caspase-independent macrophage cell death (Liu and Levine, 2015; Zhang J. et al., 2016). To determine whether NO plays a central role in IMQ-induced cell death, we assessed the production of NO^2–^ (a stable metabolite of NO⋅) in culture supernatants using the Griess reagent assay. IMQ treatment caused an increase in NO levels at an early time point (15 min) and a late time point (24 h) (Figure 2A). These increases coincided with the increased expression of iNOS at each time point (Figure 2B), suggesting that the generation of NO was a major factor in the increased levels of autophagy induced by IMQ.

The activation of mitogen-activated protein kinase (MAPK) subfamilies is important in oxidative stress-mediated cell death (Poli et al., 2004; Fulda et al., 2010). To determine whether MAPKs played a role upstream of NO generation in IMQ-induced autophagy, we first examined the activation of MAPKs by immunoblot assays using phosphospecific antibodies. The phosphorylation of MEK1/2 and ERK1/2 was detected as early as 5 min after IMQ treatment and persisted throughout the 12 h time course; however, the levels of phosphorylated p38 and JNK were detected at an early time point (Supplementary Figure S1I) but not at late time points after IMQ treatment (Figure 2C).

Activation of the PI3K/AKT signaling pathway protects cells from cell death by reducing the activity of GSK-3β (Maurer et al., 2014). While GSK-3β activity is positively regulated by phosphorylation of Tyr216, it is negatively regulated by phosphorylation of Ser9 (McCubrey et al., 2014; Beurel et al., 2015). To determine whether GSK-3β played a role upstream of NO generation in IMQ-induced autophagy, we next assessed the activity of GSK-3β. Robust activation of GSK-3β (dephosphorylation of Ser9) was detected at 3 h following exposure to IMQ, and this change was sustained for 24 h after IMQ treatment (Figure 2D). In addition, IMQ treatment increased the expression of PI3K over time and inhibited the activation of p70 S6K (phosphorylation) at late time points (12–24 h) (Figure 2D). To further characterize the role of MEK/ERK1/2 and GSK-3β as upstream kinases in the generation of NO, we pretreated cells with specific inhibitors of these kinases, and then, NO production was examined in culture supernatants. Pretreatment of cells with the MEK1/2 inhibitor U0126 significantly reduced IMQ-induced NO production at an early time point (3 h) (Figure 2E). We also found that NO production was decreased at early (3 h) and late time points (24 h) in the presence of the MEK1/2 inhibitor U0126, while it decreased at late time points (24 h; Supplementary Figure S1G) in the presence of the GSK3β inhibitor SB216763 in IMQ-treated cells. However, knockdown of GSK-3β by siRNA resulted in decreased production of NO at a late time point (24 h) in IMQ-stimulated Raw264.7 cells (Figure 2F). These results indicated that IMQ-induced NO production was modulated by the MEK/ERK1/2 pathway at the early time point and by the GSK-3β-mediated pathway at the late time point.

### IMQ Induces Autophagy by Upregulating NO Production via the MEK/ERK1/2- and GSK-3β-Mediated Pathways

**FIGURE 3:**
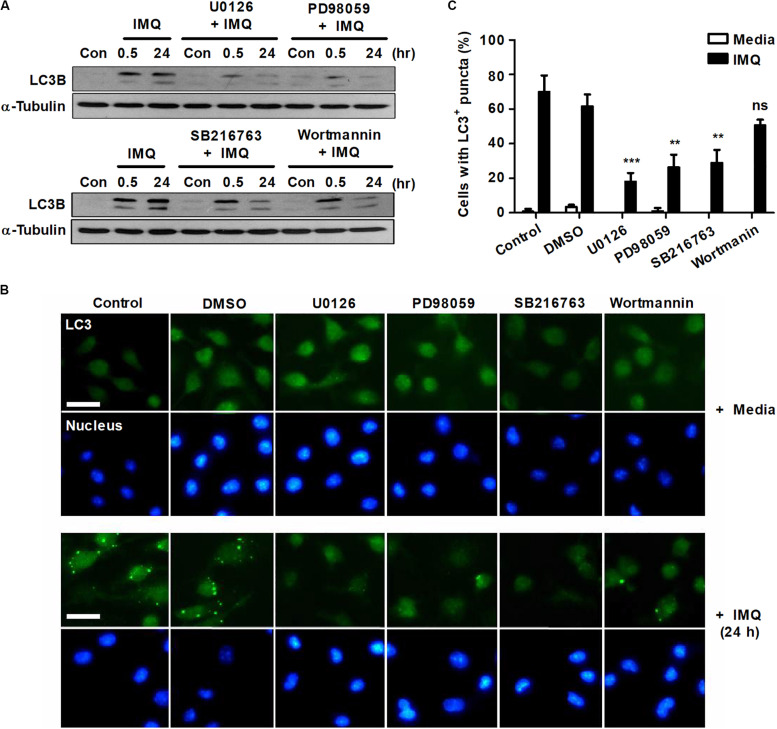
IMQ induces autophagy by enhancing NO production via MEK-ERK1/2- and GSK-3β-mediated signaling. Raw264.7 cells were pretreated with U0136 (10 μM for 2 h), SB216763 (10 μM for 2 h), PD98059 (20 μM for 1 h) or wortmannin (2 μM for 1 h) before treatment with IMQ. **(A)** Western blots were performed with antibodies against LC3 or α-tubulin. **(B)** Representative immunofluorescence images; scale bar 5 μm. **(C)** Quantitative analysis of the percentages of cells with LC3^+^ puncta at 24 h after IMQ treatment. Data are the means ± s.d. of three technical replicates and are representative of at least three independent experiments. Images are representative of at least three independent experiments. Statistical significance is indicated as **p < 0.01, ***p < 0.001 and ns, not significant (*p* > 0.05).

To determine the role of NO in the induction of autophagy, we used a range of pharmacological inhibitors to determine the role of the ERK/MEK1/2 and GSK-3β pathways in autophagy induction. Pretreatment with the MEK1 inhibitor PD98059 or the MEK1/2 inhibitor U0126 significantly reduced LC3-II expression at a late time point (24 h) as well as at an early time point (30 min) after IMQ treatment (Figure 3A). However, the inhibition of GSK-3β with SB216763 blocked the conversion of LC3-I to LC3-II only at a later time point, 24 h, in IMQ-stimulated cells (Figure 3A). To further confirm this observation, we detected endogenous LC3 by immunofluorescence staining. The percentage of cells with FITC-LC3-positive puncta was clearly reduced in IMQ-treated cells following inhibition of MEK/ERK1/2 or GSK-3β (Figures 3B,C). These findings suggested that NO production regulated by MEK/ERK1/2 and GSK-3β played an important role in the induction of autophagy in IMQ-stimulated macrophages. We also assessed the conversion from LC3-I to LC3-II in IMQ-stimulated cells pretreated with the PI3K inhibitors 3-MA (Figure 1D) or wortmannin (Figure 3A). Inhibition of PI3K using both PI3K inhibitors decreased the conversion of LC3-II in IMQ-treated cells (Figures 1D, 3A). We further demonstrated whether NO contributes to the control of intracellular Mtb growth by inducing autophagy and assessed NO production and the conversion from LC3-I to LC3-II in cells treated with sodium nitroprusside (SNP), an NO donor. Treatment with SNP promoted NO production (data not shown) as well as the conversion of LC3-II (Supplementary Figure S1J). These results suggest that NO contributes to the activation of autophagy. Although these inhibitors target the same molecules, a recent study demonstrated that 3-MA continually suppressed autophagy for a prolonged period, whereas the inhibitory effect of wortmannin was rather transient (Wu et al., 2010). In line with this study, we also confirmed that 3-MA inhibited autophagy more strongly than wortmannin.

### IMQ-Induced ROS Trigger Autophagy

**FIGURE 4:**
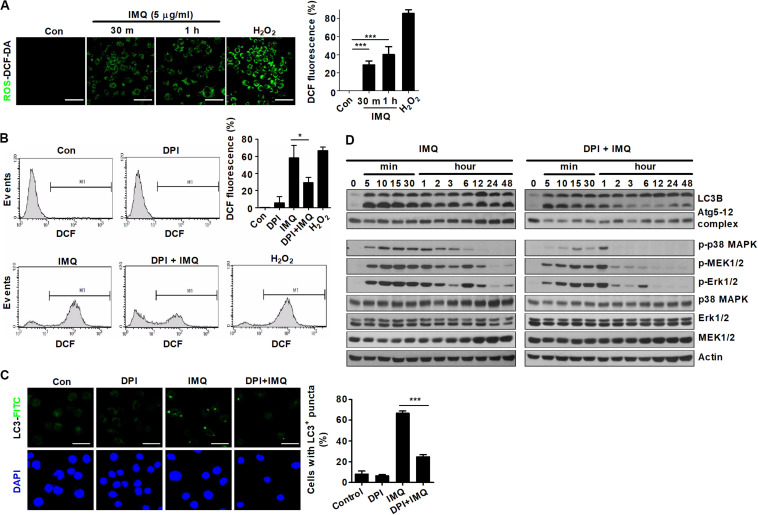
ROS are responsible for the early induction of autophagy in IMQ-treated cells. Intracellular ROS levels were determined by the intensity of DCF fluorescence using **(A)** confocal microscopy or **(B)** flow cytometry. **(A,B)** The bar graph represents the percentage of DCF fluorescence intensity. **(C,D)** Raw264.7 cells were pretreated with DPI and then treated with IMQ. LC3-positive cells were observed under confocal microscopy. The bar graph represents the percentage of cells with LC3^+^ puncta. **(D)** Western blots were performed with antibodies against each protein. Data are the means ± s.d. of three technical replicates and are representative of at least three independent experiments. Images are representative of at least three independent experiments. Statistical significance is indicated as *p < 0.05 and ***p < 0.001.

Recently, several reports have shown that autophagy is regulated by ROS, suggesting that ROS and autophagy play a central role in maintaining cellular homeostasis in response to cellular stress (Azad et al., 2009; Filomeni et al., 2015). We next assessed whether IMQ could also regulate autophagy by inducing ROS production. Treatment with IMQ strongly enhanced intracellular ROS levels at early time points; however, IMQ-induced ROS were suppressed when cells were pretreated with the NADPH oxidase inhibitor DPI (Figures 4A,B). We also demonstrated that blockade of ROS generation by DPI clearly inhibited the formation of endogenous LC3-positive puncta in IMQ-stimulated cells (Figure 4C). To examine the molecular mechanisms that trigger ROS generation in IMQ-stimulated cells, we investigated intracellular pathways via activation of various molecules associated with the MAPK signaling pathway. Inhibition of ROS generation with DPI diminished the expression of LC3-II and the Atg5-12 complex as well as the levels of phosphorylated p38 MAPK, MEK1/2 and ERK1/2 in IMQ-stimulated cells (Figure 4D). These data suggested that IMQ-induced autophagy was promoted by the upregulation of ROS generation via the MAPK signaling pathway at an early time point.

### IMQ Enhances Mitophagy by Inducing Mitochondrial ROS

**FIGURE 5:**
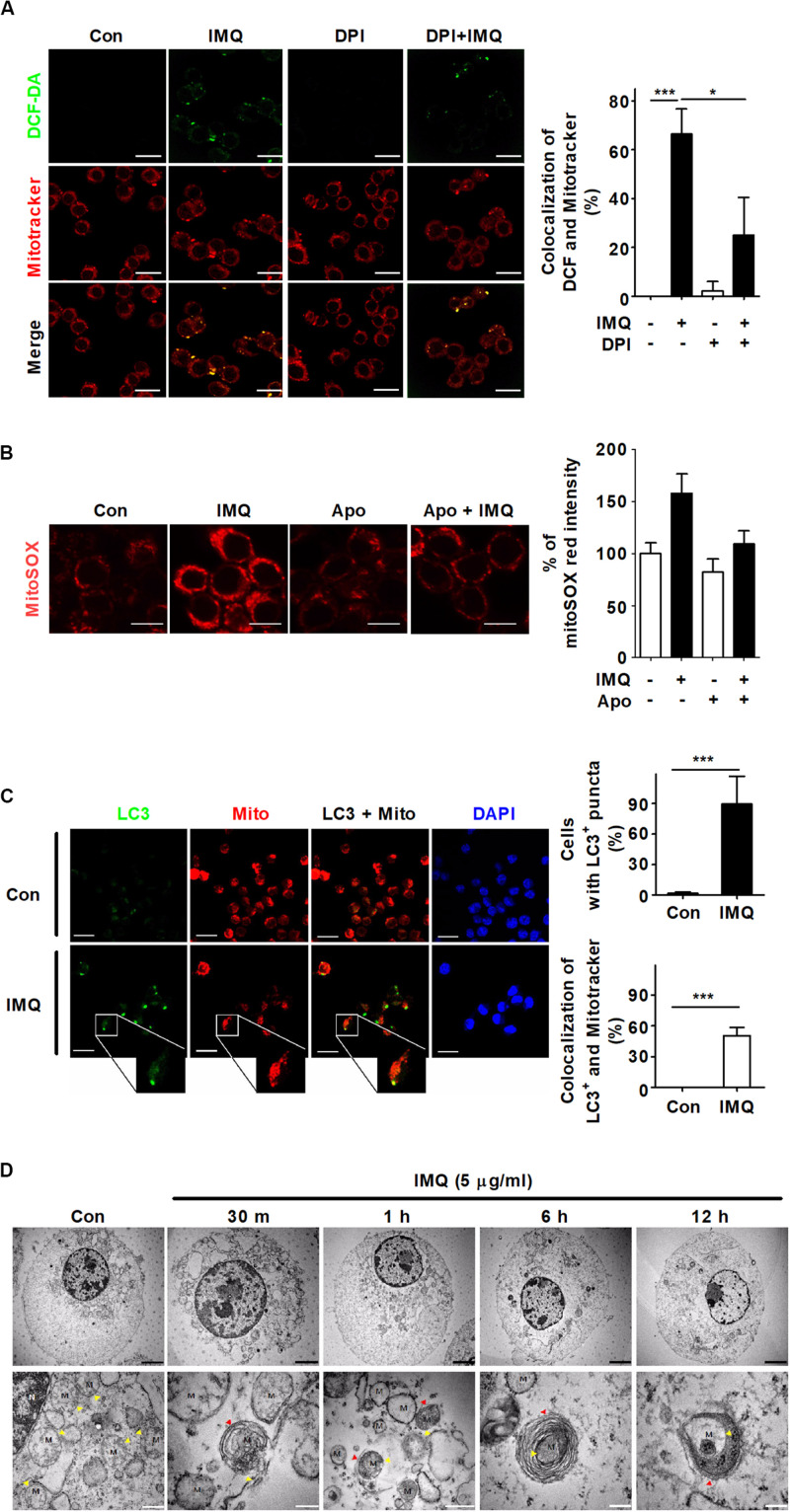
IMQ also induces mitophagy in macrophages. **(A)** Mitochondrial ROS were detected using MitoTracker Red and DCF-DA staining under confocal microscopy. The bar graph represents the percentage of MitoTracker Red overlapping with DCF fluorescence. **(B)** Raw264.7 cells were treated with IMQ in the presence or absence of an NADPH oxidase inhibitor, apocynin (Apo). Mitochondrial ROS level was measured by staining with MitoSOX Red. The bar graph represents the percentage of MitoSOX Red fluorescence intensity. **(C)** The overlapping signal of MitoTracker Red (mito) and LC3 was observed by confocal microscopy. The percentage of autophagy indicated by LC3-positive cells. The percentage of mitophagy indicates the overlapping signal between MitoTracker Red and LC3^+^ puncta. **(D)** Mitochondria surrounded by multilayer membranes were detected by TEM. Yellow arrowhead, mitochondria; red arrowhead, autophagosome; M, mitochondrion; Black scale bar, 2 μm; white scale bar, 1 μm. **(A–C)** Data are the means ± s.d. of three technical replicates and are representative of at least three independent experiments. Images are representative of at least three independent experiments. Statistical significance is indicated as *p < 0.05 and ***p < 0.001.

Several studies have shown that autophagy is regulated by ROS produced by mitochondria in mammals, indicating that ROS play important roles in autophagy (Scherz-Shouval and Elazar, 2011; Filomeni et al., 2015). A recent study showed that mild oxidative stress triggers mitophagy, which is selective autophagy and essential for maintaining mitochondrial dynamics by removing damaged or dysfunctional mitochondria (Frank et al., 2012). To demonstrate the origin of IMQ-induced ROS, we first examined mitochondrial ROS by measuring the overlapping signal of mitochondria and ROS using the probe MitoTracker Red and the ROS-sensitive dye DCF-DA. Treatment with IMQ strongly enhanced the percentage of MitoTracker Red signal overlapping with DCF fluorescence (Figure 5A). We further assessed the level of mitochondrial ROS using MitoSOX Red. The MitoSOX Red signal was significantly enhanced after IMQ treatment and attenuated by the NADPH oxidase inhibitor apocynin (Figure 5B). We investigated whether IMQ-induced mitochondrial ROS triggers mitophagy and found that IMQ treatment clearly increased the percentage of MitoTracker Red signal overlapping with LC3^+^ puncta (Figure 5C). We further obtained evidence for mitochondrial ROS-induced mitophagy in IMQ-stimulated cells. IMQ treatment increased the abundance of mitochondria surrounded by double-membrane structures, as detected by electron microscopy (Figure 5D). These data suggested that IMQ triggered mitophagy by enhancing mitochondrial ROS in macrophages.

### IMQ Induces Mitophagy via the Interaction Between Beclin-1 and BNIP3

**FIGURE 6:**
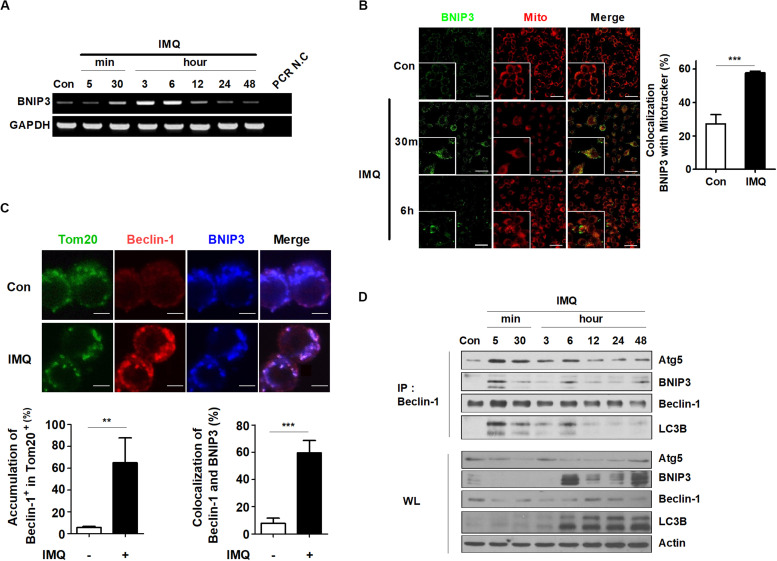
BNIP3 is recruited to the mitochondria and associated with beclin-1 in IMQ-treated macrophages. **(A)** mRNA expression of BNIP3 was detected by RT-PCR in IMQ-treated cells. **(B)** The overlapping signal between MitoTracker Red (mito) and BNIP3 was observed by confocal microscopy at 30 min and 6 h in IMQ-treated cells. The bar graph represents the percentage of MitoTracker Red overlapping with BNIP3. **(C)** Raw264.7 cells were treated with IMQ for 30 min and fixed and stained with anti-Tom20, anti-beclin-1 and anti-BNIP3 antibodies. The overlapping signal of these proteins was detected by confocal microscopy. Bar graphs represent the percentage of beclin-1 accumulation in Tom20^+^ mitochondria and beclin-1 overlapping with BNIP3. **(D)** Total cell lysates were immunoprecipitated with anti-beclin-1 and analyzed by Western blotting using antibodies against BNIP3, Atg5, beclin-1, LC3B, and actin. Data are the means ± s.d. of three technical replicates and are representative of at least three independent experiments. Images are representative of at least three independent experiments. Statistical significance is indicated as **p < 0.01 and ***p < 0.001.

Recent studies have shown that a specific type of autophagy, known as selective autophagy, plays important roles in the innate immune response against pathogens invading host cells (Gatica et al., 2018; Sharma et al., 2018). Selective autophagy can be classified according to the substrates that are sequestered by autophagosomes. Mitophagy is a type of selective autophagy that controls innate immune responses by eliminating impaired organelles and intracellular pathogens (Randow and Youle, 2014; Gkikas et al., 2018). Therefore, we determined whether IMQ controls intracellular Mtb growth by inducing selective autophagy. First, we investigated which target molecules contribute to IMQ-induced selective autophagy. BCL2 and adenovirus E1B 19-kDa-interacting protein 3 (BNIP3) is a proapoptotic mitochondrial protein that exhibits homology to Bcl2 in the BH3 domain and has death-inducing activity (Zhang and Ney, 2009). Several studies have reported that BNIP3 contributes to necrosis and autophagic cell death (Vande Velde et al., 2000; Daido et al., 2004; Azad et al., 2008). Consistent with these findings, we confirmed that IMQ-induced mitophagy was mediated by BNIP3. We first verified the BNIP3 mRNA level by using RT-PCR. The BNIP3 mRNA level was strongly increased at 30 min and 6 h after IMQ treatment compared to the control treatment. Twelve hours after IMQ treatment, BNIP3 mRNA expression returned to the basal level (Figure 6A). To assess whether BNIP3 was expressed in mitochondria, we identified the site of overlapping signal between intracellular BNIP3-FITC and MitoTracker Red by immunofluorescence staining. As shown in the representative confocal microscopy images, IMQ treatment resulted in a twofold increase in the percentage of MitoTracker Red signal overlapping with BNIP3 at 30 min and 6 h (Figure 6B). To support this hypothesis, we assessed the overlapping signal of beclin-1, BNIP3, and Tom20, which are mitochondrial outer membrane proteins. IMQ enhanced the overlapping signal of beclin-1 and BNIP3 in Tom20^+^ mitochondria (Figure 6C). A recent study showed that BNIP3 competed with beclin-1 to interact with Bcl-2 in response to hypoxia (Bellot et al., 2009), suggesting that BNIP3 can regulate mitophagy. We also questioned whether IMQ promoted mitophagy by enhancing the interaction of BNIP3 and beclin-1. Immunoprecipitation of beclin-1 was performed to evaluate the beclin-1-BNIP3 interaction by immunoblot analysis. As a result, we observed that binding of beclin-1 to BNIP3 was maximal at 5 min after IMQ treatment, and the interaction was maintained up to 48 h (Figure 6D), confirming that IMQ promoted mitophagy by enhancing the interaction between beclin-1 and BNIP3.

### IMQ-Induced Autophagy Eliminates the Intracellular Pathogen Mtb

**FIGURE 7:**
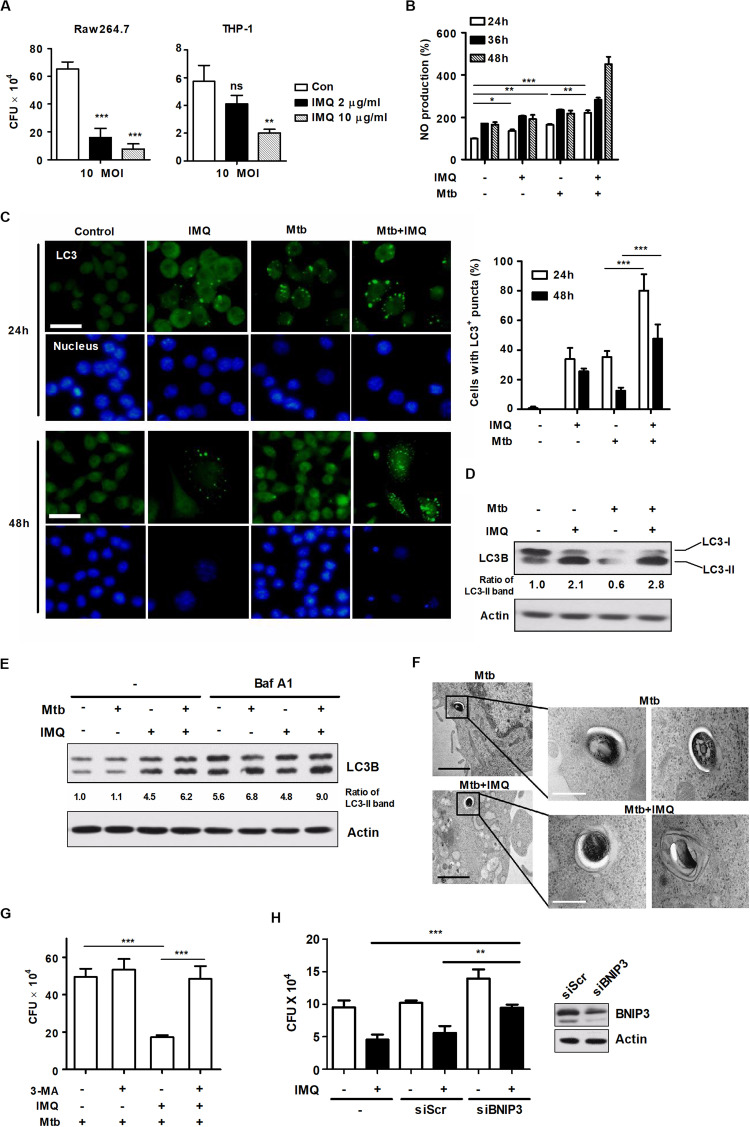
IMQ controls intracellular Mtb growth by inducing autophagy. **(A)** Raw264.7 or THP-1 cells were infected with H37Rv (at an MOI of 10) for 4 h, washed to remove extracellular bacteria and treated with IMQ (2 μg/mL, 10 μg/mL) for 3 days. Intracellular bacterial growth was measured with CFU assays. Colonies were counted at 21 days after inoculation. **(B)** Raw264.7 cells were infected with H37Rv (at an MOI of 1) for 4 h and treated with IMQ for the indicated times. NO production was detected in culture supernatants using an NO detection kit. **(C)** Intracellular LC3 expression was detected with immunofluorescence analysis under confocal microscopy. The bar graph represents the percentage of cells with LC3^+^ puncta. **(D)** Western blotting was performed with antibodies against LC3 or α-tubulin. **(E)** Raw264.7 cells were pretreated with bafilomycin A1 and then infected with H37Rv at an MOI of 1 in the presence or absence of IMQ (2 μg/mL). Protein levels of LC3B and actin were detected by a Western blot assay. **(F)** Mtb surrounded by multilayer membranes was observed by TEM. Black scale bar; 1 μm, white scale bar; 250 nm. **(G)** Raw264.7 cells were pretreated with 3-MA and then treated with IMQ after H37Rv infection. Intracellular bacterial growth was measured with CFU assays. Colonies were counted at 21 days after inoculation. **(H)** Raw264.7 cells were transfected with scrambled siRNA (siScr) or siRNA targeting BNIP3 and then infected with H37Rv at an MOI of 1 in the presence or absence of IMQ (2 μg/mL) for 48 h. Intracellular bacterial growth was assessed with a CFU assay. Data are the means ± s.d. of three technical replicates and are representative of at least three independent experiments. Images are representative of at least three independent experiments. Statistical significance is indicated as *p < 0.05, **p < 0.01, ***p < 0.001 and ns, not significant (*p* > 0.05).

*Mycobacterium tuberculosis* is an intracellular pathogen that infects and replicates in macrophages (Stutz et al., 2018). To date, published articles have shown that autophagy plays pivotal roles in host defense against Mtb (Gutierrez et al., 2004; Huang and Brumell, 2014). Recent studies have demonstrated that some PRRs, such as TLRs and NLRs, activate autophagy to promote host defense mechanisms (Oh and Lee, 2014). In accordance with these findings, we investigated whether IMQ can control intracellular Mtb survival by inducing autophagy in mouse macrophages. We first tested whether IMQ treatment can reduce the viability of mycobacteria in infected macrophages. Cells were infected with Mtb (at MOIs of 0.1, 1, and 10). Treatment with IMQ decreased intracellular Mtb survival in both macrophage cell lines at a low concentration (2 μg/mL) and a high concentration (10 μg/mL) of IMQ (Figure 7A and Supplementary Figure S2A). Our data suggest that IMQ promotes the induction of autophagy by increasing NO production in the late phase. To further verify whether NO contributes to controlling intracellular Mtb growth, Raw264.7 cells were infected with H37Rv and then treated with IMQ in the presence or absence of SNP. Combined treatment with SNP and IMQ suppressed intracellular Mtb growth compared to that in cells treated with IMQ alone (Supplementary Figure S2B). To demonstrate whether TLR7-mediated signaling is involved in the induction of autophagy, we assessed intracellular bacterial growth and intracellular LC3 levels in WT and *Myd88*^–/–^ BMDMs after IMQ treatment. The expression of intracellular LC3^+^ puncta increased in IMQ-treated WT BMDMs, whereas it was markedly decreased in IMQ-treated *MyD88*^–/–^ BMDMs (Supplementary Figure S2E). Intracellular Mtb growth did not decrease in IMQ-treated *MyD88*^–/–^ BMDMs during Mtb infection (Supplementary Figure S2D). As shown Figure 7B, NO production was significantly enhanced in IMQ-treated macrophages at 24 and 48 h postinfection. However, IMQ did not enhance TNF-α or IL-10 production during Mtb infection compared with that of cells treated with IMQ alone or infected with Mtb alone (Supplementary Figure S2F). Treatment with IMQ also strongly increased the percentage of cells with endogenous LC3-FITC punctate dots in Mtb-infected cells compared to cells infected with Mtb alone or cells treated with IMQ alone (Figure 7C). In accordance with these findings, the conversion of LC3 to LC3-II was more enhanced in IMQ-treated cells infected with Mtb than in cells infected with Mtb alone or treated with IMQ alone (Figure 7D). We further measured the overlapping signal of Mtb with LC3 and LAMP-1 by immunofluorescence. As shown in Supplementary Figure S2G, compared with infection with FITC-labeled Mtb alone, treatment with IMQ enhanced the overlapping signal of FITC-labeled Mtb with LC3 and LAMP-1, as well as LAMP-1 accumulation. We also found that the accumulation of LC3-II increased in IMQ-treated cells during Mtb infection in the presence of bafilomycin A1 (Figure 7E). To further show that IMQ enhances autophagy in Mtb-infected macrophages, we monitored Mtb-containing autophagic vacuoles by TEM. TEM analysis revealed the presence of Mtb within multiple autophagosome-like vacuoles with double-membrane structures in IMQ-treated cells (Figure 7F). We measured intracellular Mtb growth when autophagy was inhibited using 3-MA and found that inhibition of autophagy restored intracellular Mtb growth, which was reduced by IMQ treatment (Figure 7G). To determine whether BNIP3-mediated mitophagy contributes to intracellular Mtb growth, cells were transfected with siRNA targeting BNIP3. Treatment with IMQ markedly reduced intracellular Mtb growth; however, silencing BNIP3 resulted in a significant increase in intracellular Mtb growth in IMQ-treated cells (Figure 7H and Supplementary Figure S2C). These results suggested that IMQ could control intracellular Mtb growth by promoting BNIP3-mediated mitophagy. Although BNIP3 contributes to mitophagy in IMQ-treated macrophages during Mtb infection, it is still unclear how BNIP3-mediated mitophagy induced by IMQ protects Mtb-infected hosts and affects the host immune response to Mtb infection in vivo. Therefore, further research is needed to investigate the biological role of BNIP3-mediated mitophagy induced by IMQ in protecting the Mtb-infected host and enhancing host immunity to Mtb infection.

## Discussion

We showed that stimulation of TLR7 with a synthetic ligand, IMQ, induced autophagic cell death of macrophages in this study. The best-known mechanisms of cell death are apoptosis and necrosis (Green and Llambi, 2015). Another type of death is autophagy, which is intimately associated with eukaryotic cell death (Yi et al., 2009; Jorgensen et al., 2017; Woo and Jung, 2017). Autophagy constitutes a stress adaptation pathway that promotes cell survival under most circumstances, including starvation, chemotherapeutic agents or other toxic compounds (Levine and Kroemer, 2008; Fulda et al., 2010). In addition, autophagy plays a role in innate and adaptive immunity as a mechanism for the elimination of intracellular bacteria, including Mtb, *Listeria monocytogenes*, *Streptococcus pyogenes*, *Salmonella*, and *Shigella* (Xu and Eissa, 2010; Yano and Kurata, 2011). However, autophagy is also considered a type of nonapoptotic programmed cell death called “type II” or “autophagic” cell death (Liu and Levine, 2015). The factors that determine whether autophagy is cytoprotective or cytotoxic and how autophagy induces cell death are still unclear. In this study, we found that inhibition of autophagy with 3-MA provided cellular protection from IMQ-induced cell death and suppressed the mobilization of LC3, which was increased in IMQ-treated cells, suggesting that IMQ induced autophagic cell death in macrophages.

Our results suggested that the connections between autophagy and TLR signaling were linked to broad aspects of innate immunity. In other words, the present study elucidated TLR signaling-induced autophagy activation, which might contribute to antimycobacterial immunity, through the observation that autophagy induction in response to IMQ, a TLR7 agonist, eliminated intracellular pathogens in macrophages. Consistent with these results, MA (Delgado et al., 2008) reported that a subset of TLR ligands, such as poly(I:C) (TLR3), LPS (TLR4) and ssRNA (TLR7), induced autophagy in murine macrophages.

Although imidazoquinoline derivatives are known to be TLR7 agonists, IMQ is the only TLR7 agonist that has been developed as a medical drug targeting TLR7 to date. IMQ is used to treat human papillomavirus (Edwards et al., 1998; Chen, 2013), herpes simplex virus (Hirokawa et al., 2011), and cancer (Geisse et al., 2004). It has been suggested that IMQ has potential as a treatment for cutaneous breast cancer (Kohrt, 2012). Han et al. (2013) showed that IMQ suppressed the growth of prostate cancer cells in human and mice by inducing cell cycle arrest. In addition, it is already known that IMQ has a very weak TLR7 binding affinity compared to other TLR7 agonists because it lacks an R1-mediated hydrophobic interaction (Zhang et al., 2018). Hood et al. (2010) have shown that compared to GDQ, IMQ reduced NF-κB luciferase activity at specific concentrations. Consistent with these studies, Gross et al. (2016) found that IMQ induced IL-1β by activating the NLRP3 inflammasome regardless of TLR7, even though IMQ is an agonist of TLR7. Walter et al. (2013) also demonstrated that Aldara, a pharmacologic cream containing 5% IMQ, triggered inflammation through the production of cytokines, which was independent of TLR7. They found that treatment with Aldara enhanced the production of inflammatory cytokines, including IL-1β, IL-6, and TNF, in keratinocytes that do not express TLR7. These studies suggest that IMQ does not require TLR7 to induce inflammation. However, we found that IMQ induced a selective autophagy via TLR7-MyD88-mediated signaling, according to our results demonstrating decreased expression of intracellular LC3^+^ puncta in IMQ-treated *MyD88*^–/–^ BMDMs, although there was no direct evidence for the requirement of TLR7 in the induction of selective autophagy. Therefore, further studies are needed to clarify whether the involvement of TLR7 in BNIP3-mediated selective autophagy and inflammasome activation affects innate defense mechanisms against Mtb infection.

Lee et al. (2007) showed that autophagy contributed to delivery of a viral TLR7 ligand to the intracellular compartment where TLR7 is localized and that engagement between TLR7 ligands and TLR7 initiated signaling, which led to antiviral responses. This report demonstrated that autophagy might enhance TLR recognition of PAMPs, suggesting that autophagy regulates TLR signaling.

How do TLRs induce autophagy? The correlation between TLR signaling and autophagy has two possible explanations. First, it has been suggested that the TLR signaling pathway directly activates autophagy via TLR signaling downstream molecules. Sanjuan et al. (2007) showed that TLR signaling links the autophagy pathway to phagocytosis through TLR2 ligand-induced recruitment of Atg5, Atg7, and PI3K to the phagosome to form autophagosomes. Delgado et al. (2008) also reported that TLR7 engagement induces autophagy and controls the intracellular bacterium BCG in a MyD88-dependent manner. These reports showed that TLRs have an intrinsic ability to induce autophagy. Second, TLR signaling indirectly induces autophagy via messenger molecules produced by TLR signaling, demonstrating that it acts as an autophagy activator. Recently, several groups reported that excessive ROS and NO function as signaling molecules in a variety of intracellular processes, and they might also contribute to caspase-independent macrophage cell death (Liu and Levine, 2015; Zhang J. et al., 2016). Consistent with these findings, our results showed that IMQ treatment caused increases in NO and ROS levels, indicating that both molecules could play an important role in the induction of autophagy in IMQ-treated macrophages. Recently, Gong et al. (2012) found that tetrandrine isolated from the Chinese medicinal herb *Stephania tetrandra* induced autophagy, which was dependent on the increase in intracellular ROS accumulation following mitochondrial dysfunction and the activation of ERK1/2, a downstream molecule of MEK1/2, in human hepatocellular carcinoma cells. Similarly, our results showed that IMQ treatment triggered autophagy through an increased level of intracellular ROS via MEK1/2-mediated signaling at an early time point. Chang et al. (2013) also demonstrated that Group A Streptococcus (GAS) activated GSK-3β, which induced NO production by activating NF-κB in a mouse macrophage cell line, suggesting that GSK-3β could regulate NO production. Our previous study found that activation of GSK-3β resulted in a decrease in proinflammatory cytokine secretion via inhibition of the NF-κB signaling pathway in lysophosphatidylcholine (LPC)-treated cells during Mtb infection (Lee et al., 2018). As in the above study, we found that NO production increased in IMQ-treated cells and induced autophagy through GSK-3β-mediated signaling at a late time point. Our results indicate that autophagy is triggered by ROS and NO produced at various time intervals through TLR signaling.

Autophagy plays a role in innate immunity against intracellular pathogens by eliminating microbes directly via ingestion into autophagosomes. Our previous study demonstrated that intracellular Mtb growth was controlled in pasakbumin A-treated macrophages by enhancing autophagy via the ERK-mediated pathway (Lee et al., 2019), indicating that autophagy serves as a defense mechanism against Mtb infection in macrophages. Bao et al. (2017) found that treatment with the TLR7 agonist ssRNA induced autophagy and regulated cell viability during Mtb infection, indicating that activation of TLR7 could control Mtb infection via autophagy. However, the molecular mechanisms induced by TLR7 activation have remained unexplored. In this study, we found that treatment with IMQ increased the expression of the mitophagy-related protein BNIP3, which is located in mitochondria. Furthermore, treatment with IMQ increased the overlapping signal between LC3 and mitochondria surrounded by multilayer membranes over time. A recent study showed that the ubiquitin ligase parkin, which plays a role in the autophagic targeting of dysfunctional mitochondria (mitophagy), increases susceptibility to infection with intracellular bacteria, such as Mtb, *Mycobacterium leprae*, and *Salmonella typhimurium* (Manzanillo et al., 2013). These results suggest that mitophagy-related molecules can enhance the susceptibility to intracellular pathogens. Although a recent study showed that mitophagy is involved in the control of viral or bacterial infection (Kirienko et al., 2015; Baldanta et al., 2017; Gkikas et al., 2018), whether BNIP3 can control bacterial infection is not known. We also found that BNIP3 was associated with the elimination of Mtb through the interaction with beclin-1 in IMQ-treated macrophages. These findings suggest that IMQ could control intracellular bacterial survival by enhancing mitophagy through the interaction between the mitophagy-related protein BNIP3 and beclin-1 in macrophages. Collectively, our present study demonstrates novel mechanisms for IMQ-induced selective autophagy that exert antimicrobial activity and provides new insights into novel approaches to control Mtb infection.

## Data Availability Statement

All datasets generated in this study are included in Supplementary Data Sheet 1.

## Author Contributions

Y-JJ and H-JL conceived the study and designed the research. H-JL, S-JK, and H-JK performed the experiments and analyzed the data. H-JL and Y-JJ wrote the manuscript with input from the other authors. YW, H-JK, and T-WH contributed with materials and analysis tools. All authors contributed to the article and approved the submitted version.

## Conflict of Interest

The authors declare that the research was conducted in the absence of any commercial or financial relationships that could be construed as a potential conflict of interest.
