# Supplementary material for: TLR7 Stimulation With Imiquimod Induces Selective Autophagy and Controls Mycobacterium tuberculosis Growth in Mouse Macrophages
Source: Front Microbiol. 2020 Jul 17;11:1684. doi: 10.3389/fmicb.2020.01684 (PMC7380068; doi:10.3389/fmicb.2020.01684)
Supplement: Supplementary file 2 [file Data_Sheet_2.docx]

Supplementary Material

TLR7 stimulation with imiquimod induces selective autophagy and controls *Mycobacterium tuberculosis* growth in mouse macrophages

Hyo-Ji Lee^1,2^, Su-Jin Kang^1^, Yunseo Woo^1,2^, Tae-Wook Hahn^3^, Hyun-Jeong Ko^4^

and Yu-Jin Jung^1,2*^

*** Correspondence:**

Corresponding Author :

Yu-Jin Jung

[yjjung@kangwon.ac.kr](mailto:yjjung@kangwon.ac.kr)


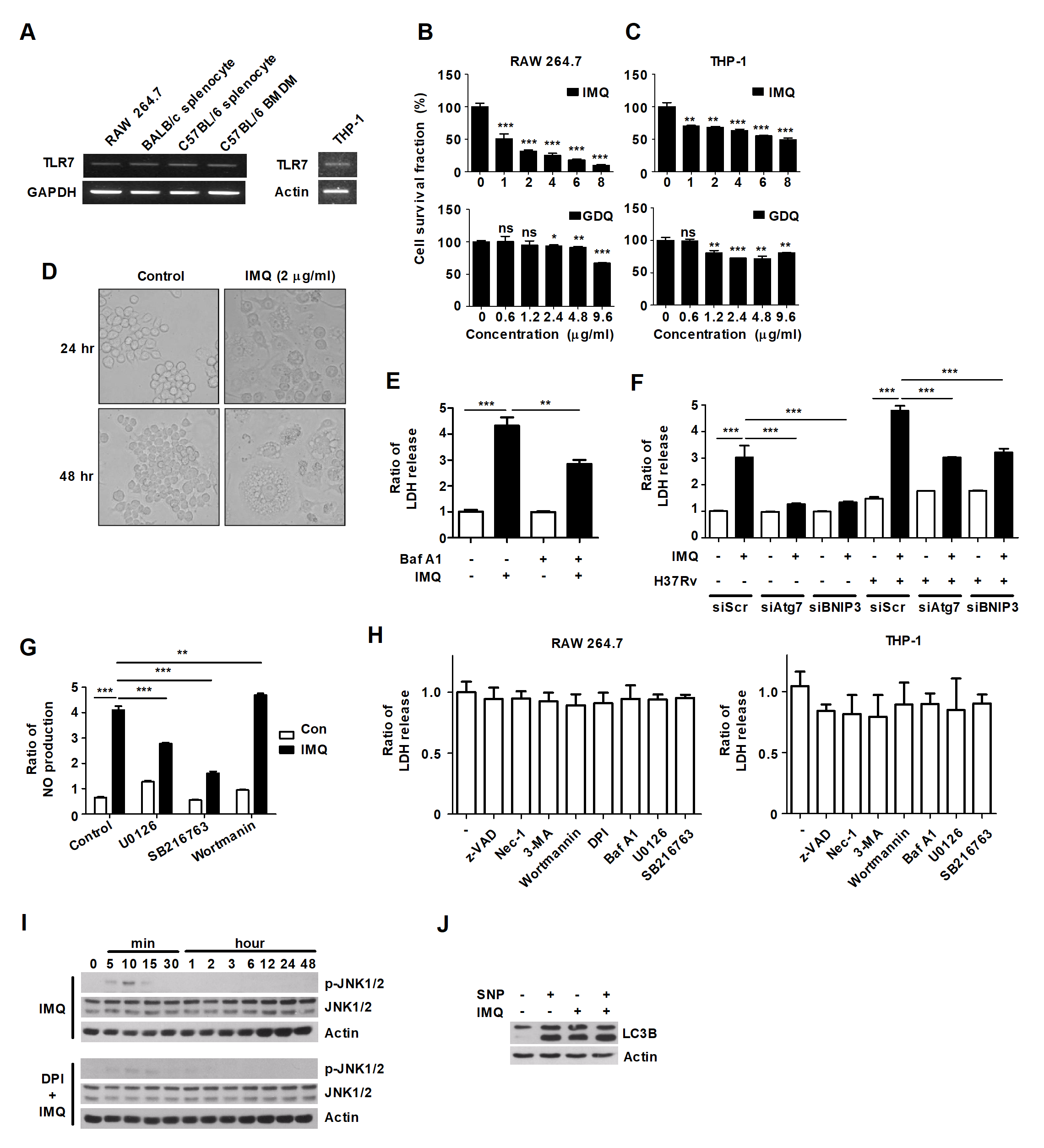


**Supplementary Figure S1.** **IMQ induces autophagy by increasing NO and mitochondrial ROS production.** (A) mRNA expression of TLR7 was detected by RT-PCR in Raw264.7 cells, BALB/c splenocytes, C57BL/6 splenocytes, C57BL/6 BMDMs and THP-1 cells. Raw264.7 (B) or THP-1 (C) cells were treated with the various concentrations of IMQ (1 μg/ml to 8 μg/ml) or GDQ (0.6 μg/ml to 9.6 μg/ml) for 48 h, and cell survival was assessed by the WST-1 assay. (D) Raw264.7 cells were treated with IMQ, and cell morphology was observed under microscopy. (E) Raw264.7 cells were pretreated with bafilomycin A1 (Baf A1) for 1 h and then treated with IMQ for 48 h. Cell viability was measured by LDH release. (F) Raw264.7 cells were transfected with siRNA (siScr), siRNA targeting Atg7 or BNIP3. Cells were infected with H37Rv and then treated with IMQ for 48 h. Cell viability was measured by LDH release. (G) Raw264.7 cells were pretreated with U0126, SB216763, or wortmannin for 1 h and then treated with IMQ for 24 h. Culture supernatants were used for the detection of NO production. (H) Raw264.7 cells were pretreated with indicated inhibitors for 1 h and then incubated for 48 h. Cell viability was measured by LDH release. (I) Raw264.7 cells were pretreated with DPI and then treated with IMQ. Western blots were performed with antibodies to p-JNK1/2, JNK1/2 and actin. (J) Raw264.7 cell were treated with SNP alone, IMQ alone or IMQ and/or SNP for 48 h. Western blots were performed with antibodies to LC3 actin in Raw264.7 cells. Data are the means ± s.d. of three technical replicates and are representative of two independent experiments. Images are representative of two independent experiments. Statistical significance is indicated as *, *p*<0.05, **, *p*<0.01, ***, *p*<0.001 and ns, not significant (p>0.05).
