# Supplementary material for: TLR7 Stimulation With Imiquimod Induces Selective Autophagy and Controls Mycobacterium tuberculosis Growth in Mouse Macrophages
Source: Front Microbiol. 2020 Jul 17;11:1684. doi: 10.3389/fmicb.2020.01684 (PMC7380068; doi:10.3389/fmicb.2020.01684)
Supplement: Supplementary file 3 [file Data_Sheet_3.docx]

Supplementary Material

TLR7 stimulation with imiquimod induces selective autophagy and controls *Mycobacterium tuberculosis* growth in mouse macrophages

Hyo-Ji Lee^1,2^, Su-Jin Kang^1^, Yunseo Woo^1,2^, Tae-Wook Hahn^3^, Hyun-Jeong Ko^4^

and Yu-Jin Jung^1,2*^

*** Correspondence:**

Corresponding Author :

Yu-Jin Jung

[yjjung@kangwon.ac.kr](mailto:yjjung@kangwon.ac.kr)


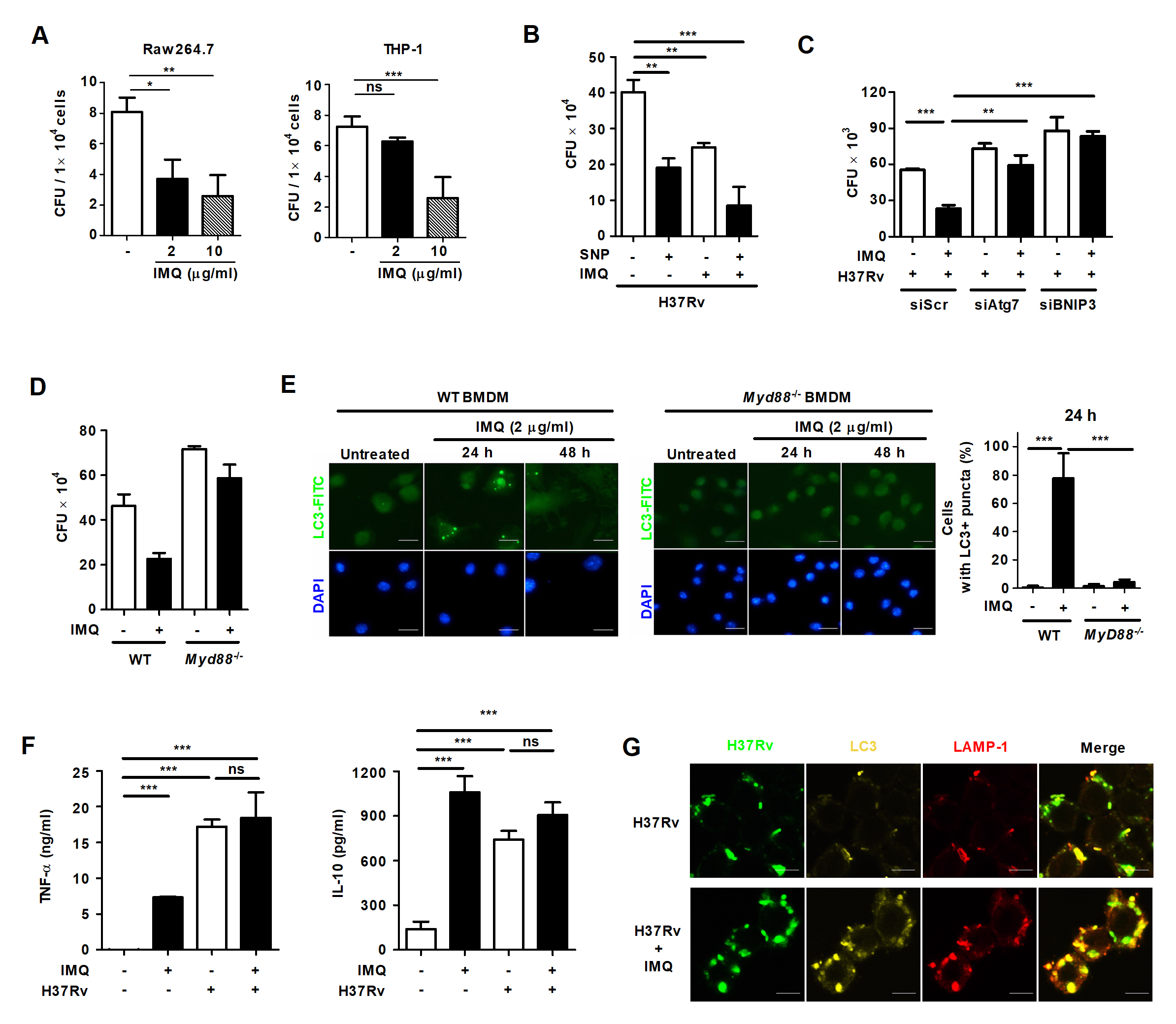


**Supplementary Figure S2. IMQ controls intracellular Mtb growth.** (A) Raw264.7 or THP-1 cells were infected with H37Rv at an MOI of 10 for 4 h and then treated with IMQ (1 or 10 μg/ml) for 3 days. Intracellular bacterial growth was measured by a CFU assay and calculated as CFU per 1 ⅹ 10^4^ cells. (B) Raw264.7 cells were infected with H37Rv and then treated with SNP alone, IMQ alone or IMQ and/or SNP for 48 h. Intracellular bacterial growth was measured with CFU assays. (C) Raw264.7 cells were transfected with siRNA (siScr), siRNA targeting Atg7 or BNIP3. Cells were infected with H37Rv and then treated with IMQ for 48 h. Intracellular bacterial growth was measured with CFU assays. (D) WT or *Myd88*^-/-^ BMDMs were infected with H37Rv and then treated with IMQ for 48 h. Intracellular bacterial growth was measured with CFU assays. Colonies were counted at 21 days after inoculation. Data are the means ± s.d. of three technical replicates. (E) WT or *Myd88*^-/-^ BMDMs were treated with IMQ for the indicated times. Intracellular LC3 expression was detected with immunofluorescence analysis under confocal microscopy. The bar graph represents the percentage of cells with LC3^+^ puncta. (F) Raw264.7 cells were treated with IMQ for 48 h, and TNF-α and IL-10 production in the culture supernatant was determined by ELISA. (G) Raw264.7 cells were infected with FITC-labeled H37Rv and then treated with IMQ for 3 h. The cells were fixed and stained with anti-LC3 and anti-LAMP-1. The overlapping signal of these proteins was detected by confocal microscopy. Data are the means ± s.d. of three or four technical replicates and are representative of two independent experiments. Images are representative of two independent experiments. Statistical significance is indicated as **, *p*<0.01, ***, *p*<0.001 and ns, not significant (*p*>0.05).
